# Role of Age and Acculturation in Diet Quality Among Mexican Americans — Findings From the National Health and Nutrition Examination Survey, 1999–2012

**DOI:** 10.5888/pcd14.170004

**Published:** 2017-07-20

**Authors:** Yilin Yoshida, Richard Scribner, Liwei Chen, Stephanie Broyles, Stephen Phillippi, Tung-Sung Tseng

**Affiliations:** 1 Department of Health Management and Informatics, School of Medicine, University of Missouri, Columbia, Missouri; 2 Epidemiology, School of Public Health, Louisiana State University Health Sciences Center at New Orleans, New Orleans, Louisiana; 3 Department of Public Health Sciences, Clemson University, Clemson, South Carolina; 4 Behavioral and Community Health Sciences, School of Public Health, Louisiana State University Health Sciences Center at New Orleans, New Orleans, Louisiana; 5 Pennington Biomedical Research Center, Baton Rouge, Louisiana

## Abstract

Age and acculturation may play a role in diet quality among Mexican Americans. This study examined diet quality in Mexican Americans by age and whether acculturation influences diet quality across different age groups, using data from the National Health and Nutrition Examination Survey (NHANES). Diet quality, measured by the Healthy Eating Index 2010, improved with age except in categories of dairy, sodium, and refined grains. More acculturation was associated with lower scores in overall diet quality and categories of vegetables, fruits, and sodium and empty calories across almost all ages, but higher scores in grain categories, especially in younger groups. A diet rich in fruits and vegetables but low in fat and sodium should be promoted among more acculturated Mexican Americans, and whole-grain foods should be promoted among young but less acculturated Mexican Americans.

## Objective

In Mexican Americans, diet quality is linked with various clinical outcomes (1,2), and it may vary by age. Young Mexican Americans are more likely to consume nutrition-poor but energy-dense foods compared with their older counterparts (3). Although the diet of older Mexican Americans is usually healthy, their diets may be insufficient in recommended nutrients (4). Additionally, acculturation — a process by which groups adopt the customs and behaviors of a new culture — may further complicate nutrition status, because acculturation is associated with adverse nutritional profiles (5). To help design dietary interventions for Mexican Americans, we sought to understand the potential age differences in diet quality and whether acculturation influences diet quality overall or in specific aspects across different ages.

## Methods

We used data from the National Health and Nutrition Examination Survey (NHANES) cycles 1999–2000 through 2010–2012 (6). Data from the US Department of Agriculture Food Patterns Equivalents Database were obtained to translate NHANES 24-hour dietary recall data into equivalent servings of the major food groups according to the Healthy Eating Index (HEI) 2010 (7). The National Center for Health Statistics Research Ethics Review Board approved NHANES. Informed consent was obtained from all participants (6).

Acculturation was constructed as an acculturation index (0 = least acculturated to 5 = most acculturated) derived from measures of country of birth, language spoken at home, and length of time in the United States (8). Diet quality was measured by the HEI 2010, which contains 12 categories: 9 “adequacy” categories (total vegetables, greens and beans, total fruit, whole fruit, total proteins, seafood and plant proteins, whole grains, dairy, and fatty acids) and 3 “moderation” categories (refined grains, sodium, and empty calories). Moderation components were reverse-scored. These scores were summed to yield a score ranging from 1 to 100, with a higher score indicating a better diet quality (7). Covariates included sex, education level, marital status, poverty-to-income ratio (the ratio of total family income to the poverty threshold for a family of that size), insurance coverage, smoking status, and alcohol drinking status. Age groups were categorized as young adults (aged 20–39 y), middle-aged adults (aged 40–60 y) and seniors (aged >60 y).

Analysis of variance and χ^2^ tests were used to examine the differences of HEI scores and sociodemographic characteristics across age groups. Because of nonnormal distributions of HEI scores, each score was dichotomized based on median values. Scores above the median reflect better dietary intakes. Multiple logistic regressions were performed to investigate the association between acculturation and diet quality across age groups. All analyses were conducted using SAS version 9.4 (SAS Institute, Inc), adjusting for complex survey design effects.

## Results

The overall diet quality (ie, HEI total) significantly increased with increasing age (Table 1). Older Mexican Americans had higher scores in nearly all HEI component categories except for dairy, sodium, and refined grains, for which young adults had higher scores compared with other age groups. Acculturation and other sociodemographic characteristics varied significantly by age as well, as seniors were more acculturated (mean scores were 2.9 for seniors, 2.8 for middle-aged adults, and 2.2 for young adults) and had higher proportions of attaining education less than high school, of living in poverty, and of having public insurance coverage but lower proportions of current smokers or drinkers, compared with young or middle-aged adults.

**Table 1:** Healthy Eating Index 2010 Scores and Sociodemographic Characteristics of Mexican Americans by Age (N = 6,847), National Health and Nutrition Examination Survey (NHANES), 1999–2000 Through 2010–2012^a^

Category	Young Adults (Aged 20–39 y) (n = 3,096)	Middle-Aged Adults (Aged 40–60 y) (n = 1,948)	Seniors (Aged >60 y) (n = 1,803)	*P*^b^
**Healthy Eating Index 2010 Category (Maximum Score)^c^, Mean (SE)**				
**Total score (100)**	48.46 (0.34)	50.92 (0.43)	53.90 (0.58)	<.001
Total vegetables (5)	3.44 (0.03)	3.60 (0.04)	3.64 (0.05)	<.001
Greens and beans (5)	2.01 (0.05)	2.17 (0.07)	2.26 (0.07)	<.001
Total fruit (5)	2.00 (0.05)	2.20 (0.07)	2.54 (0.07)	<.001
Whole fruit (5)	1.79 (0.06)	2.22 (0.07)	2.56 (0.07)	<.001
Whole grains (10)	1.14 (0.05)	1.57 (0.08)	2.39 (0.11)	<.001
Dairy (10)	4.62 (0.09)	4.56 (0.08)	4.56 (0.09)	<.001
Total proteins (5)	4.36 (0.02)	4.48 (0.03)	4.34 (0.03)	<.001
Seafood and plant proteins (5)	1.88 (0.04)	2.14 (0.06)	2.03 (0.07)	<.001
Fatty acids (10)	5.09 (0.08)	5.30 (0.09)	5.34 (0.10)	<.001
Sodium (10)	5.47 (0.09)	5.29 (0.10)	5.14 (0.11)	<.001
Refined grains (10)	4.70 (0.09)	4.58 (0.11)	4.24 (0.15)	<.001
Empty calories (20)^d^	11.92 (0.17)	12.80 (0.58)	14.20 (0.20)	<.001
**Acculturation^e^, Mean (SE)**				
**Index score**	2.2 (0.09)	2.8 (0.05)	2.9 (0.07)	<.001
**Sociodemographic characteristics, %**				
**Sex**				
Male	54.5	51.7	55.9	<.001
**Education level**				
Less than high school graduate	48.2	56.7	72.8	<.001
High school graduate or equivalent	23.8	17.0	11.8	
More than high school graduate	28.0	26.3	15.4	
**Poverty-to-income ratio^f^**				
<1	33.3	25.2	36.7	<.001
1 to <3	48.3	46.7	48.0	
≥3	18.4	28.1	15.3	
**Marital status**				
Married	51.7	67.2	55.9	<.001
**Health insurance**				
None	58.7	44.0	24.0	<.001
Public	8.5	12.1	55.8	
Private	32.8	43.9	20.2	
**Smoking status**				
Nonsmoker	65.7	55.3	52.4	<.001
Former	13.4	24.7	34.0	
Current	20.9	20.0	13.6	
**Alcohol drinking status**				
Nondrinker	13.6	15.0	26.4	<.001
Former	13.2	21.0	35.4	
Current	73.2	64.0	38.2	

Abbreviation: SE, standard error.

a All results are weighted.

b For the Healthy Eating Index and acculturation scores, analysis of variance test was used; for sociodemographic characteristics, χ^2^ test was used.

c A higher score means a better-quality diet.

d Empty calories are from solid fats, alcohol, and added sugars.

e Acculturation score was derived from information on length of stay, nativity, and language spoken at home. Combining country of birth and length of time in the United States, a 0–3 score was assigned based on 4 categories (3 = US-born, 2 = foreign-born and lived in the United States ≥20 years, 1 = foreign-born and lived in the United States 10–19 years, 0 = foreign-born and lived in the United States <10 years). A score of 0–2 was assigned to language spoken at home (2 = English only or prefer to speak English at home, 1 = both equally, 0 = Spanish or prefer to speak Spanish at home). These scores were then summed to yield a total acculturation score, ranging from 0 (least acculturated) to 5 (most acculturated).

f Calculated as the ratio of total family income to the poverty threshold for a family of that size.

In adjusted analysis, more acculturation was significantly associated with lower HEI total score across all ages: odds ratio (OR), 0.87 (95% confidence interval [CI], 0.82–0.93) for young adults; OR, 0.90 (95% CI, 0.83–0.97) for middle-aged adults; and OR, 0.79 (95% CI, 0.71–0.88) for seniors (Table 2). With respect to HEI components, a 1-unit increase of acculturation was associated with 10% to 20% lower odds of attainting better scores for vegetables, fruits, dairy, sodium, and empty calories in almost all ages. More acculturation was also associated with a lower proteins score in young adults (OR, 0.92; 95% CI, 0.85–0.99). Opposing findings occurred in categories of whole and refined grains, where more acculturation was associated with better scores, especially in young adults for whole grains (OR, 1.13; 95% CI, 1.07–1.20) and refined grains (OR, 1.20; 95% CI, 1.15–1.25).

**Table 2:** Logistic Regression Models for the Association Between Acculturation and Diet Quality, by Age, Among Mexican Americans, National Health and Nutrition Examination Survey (NHANES), 1999–2000 Through 2010–2012^a^

Component	Young Adults (Aged 20–39 y)		Middle-Aged Adults (Aged 40–60 y)		Seniors (Aged >60 y)	
	Unadjusted OR (95% CI)^b^	Adjusted OR (95% CI)^b^^,^ c	Unadjusted OR (95% CI)^b^	Adjusted OR (95% CI)^b^^,^ c	Unadjusted OR (95% CI)^b^	Adjusted OR (95% CI)^b^^,^ c
**Total HEI 2010 score**	0.91 (0.86–0.95)	0.87 (0.82–0.93)	0.92 (0.88–0.97)	0.90 (0.83–0.97)	0.90 (0.84–0.97)	0.79 (0.71–0.88)
Total vegetables	0.91 (0.87–0.95)	0.90 (0.84–0.96)	0.86 (0.80–0.91)	0.86 (0.78–0.95)	0.98 (0.90–1.07)	0.94 (0.84–1.05)
Greens and beans	0.87 (0.83–0.92)	0.85 (0.79–0.90)	0.84 (0.79–0.90)	0.87 (0.79–0.94)	0.88 (0.83–0.95)	0.88 (0.80–0.97)
Total fruit	0.84 (0.80–0.88)	0.82 (0.77–0.87)	0.91 (0.86–0.98)	0.87 (0.79–0.96)	0.91 (0.86–0.96)	0.81 (0.75–0.88)
Whole fruit	0.88 (0.85–0.91)	0.83 (0.79–0.87)	0.91 (0.86–0.97)	0.83 (0.75–0.92)	0.92 (0.86–0.98)	0.79 (0.72–0.88)
Whole grain	1.25 (1.20–1.30)	1.13 (1.07–1.20)	1.24 (1.18–1.31)	1.07 (1.00–1.16)	1.19 (1.08–1.31)	1.07 (0.94–1.22)
Dairy	1.02 (0.97–1.07)	0.97 (0.92–1.03)	0.93 (0.88–0.98)	0.91 (0.83–0.99)	0.82 (0.75–0.88)	0.80 (0.73–0.88)
Total proteins	0.91 (0.86–0.96)	0.92 (0.85–0.99)	0.95 (0.88–1.03)	0.95 (0.89–1.02)	0.97 (0.91–1.05)	1.01 (0.91–1.13)
Seafood and plant protein	1.04 (0.98–1.09)	1.00 (0.93–1.08)	1.05 (0.98–1.12)	1.00 (0.93–1.09)	1.01 (0.95–1.05)	0.98 (0.89–1.09)
Fatty acids	0.97 (0.92–1.02)	0.96 (0.90–1.02)	0.99 (0.94–1.05)	0.98 (0.90–1.08)	0.99 (0.90–1.08)	0.97 (0.87–1.07)
Sodium	0.88 (0.83–0.91)	0.87 (0.83–0.92)	0.87 (0.83–0.92)	0.89 (0.84–0.95)	0.81 (0.75–0.88)	0.82 (0.71–0.93)
Refined grains	1.15 (1.09–1.23)	1.20 (1.15–1.25)	1.21 (1.15–1.27)	1.10 (0.99–1.21)	1.18 (1.07–1.31)	1.10 (0.97–1.26)
Empty calories	0.90 (0.87–0.94)	0.89 (0.84–0.93)	0.93 (0.89–0.98)	0.94 (0.87–1.01)	0.91 (0.85–0.99)	0.89 (0.79–0.99)

Abbreviations: CI, confidence interval; HEI, Healthy Eating Index 2010; OR, odds ratio.

a HEI total and component scores were dichotomized based on median values.

b Odds of better diet quality (ie, score above median) with 1-unit increase of acculturation.

c Adjusted for age, income, education level, marital status, alcohol drinking status, and smoking status. Acculturation was derived from information on length of stay, nativity, and language spoken at home.

## Discussion

Similar to the diet quality of the general population, diet quality for Mexican Americans increased with increasing age (2,9). Such improvement reflects a changing health consciousness as people age (9). Opposite results were observed in categories of dairy, sodium, and refined grains, where the scores decreased with increasing age. Despite the increased consumption of whole grains, refined grains may be the traditional source of carbohydrates for and are preferably consumed by older Mexican Americans (10). This inverse relationship between dairy score and age may be partially due to older people’s misconception about lactose intolerance, which may result in lower dairy intakes (11). Regarding the association between sodium and age, previous research suggested mixed results (10,12). One study found older adults had lower HEI 2005 sodium scores than did their younger counterparts (10). However, others reported that absolute sodium intake decreases with age among US adults including Mexican Americans (12). Older Mexican Americans adults may consume Mexican American foods that contains high levels of sodium because of overseasoning (12). Additional research is warranted to better understand sodium intake by age in Mexican Americans.

More acculturation was associated with poorer diet quality overall and in specific aspects across most age groups of Mexican Americans, which was in line with prior work demonstrating the negative influence of acculturation on dietary changes (5). More acculturation was positively associated with higher scores for whole and refined grains. However, this relationship was not significant in seniors. Perhaps the preference of white rice and refined wheat flour products persisted among older Mexican Americans (10). In comparison, young adults may be more open to the wider varieties of whole-grain products that the US food environment offers. This may explain the positive association between acculturation and grain scores in younger groups.

Diet quality differed by age in Mexican Americans, and acculturation contributed to variations in diet quality. The benefits of dairy and whole grains intakes should be emphasized among older Mexican Americans. A nutrition-balanced diet that is rich in fruits and vegetables but low in fat and sodium should be promoted among those who are more acculturated, whereas whole-grain foods should be further promoted among young but less-acculturated Mexican Americans.

## References

[1] Yoshida Y , Scribner R , Chen L , Broyles S , Phillippi S , Tseng TS . Diet quality and its relationship with central obesity among Mexican Americans: findings from National Health and Nutrition Examination Survey (NHANES) 1999–2012. Public Health Nutr 2017;20(7):1193–202. 27974064 10.1017/S1368980016003190 PMC10261487

[2] Guo X , Warden BA , Paeratakul S , Bray GA . Healthy Eating Index and obesity. Eur J Clin Nutr 2004;58(12):1580–6. 10.1038/sj.ejcn.1601989 15162130

[3] Hiza HA , Casavale KO , Guenther PM , Davis CA . Diet quality of Americans differs by age, sex, race/ethnicity, income, and education level. J Acad Nutr Diet 2013;113(2):297–306. 10.1016/j.jand.2012.08.011 23168270

[4] Gregory-Mercado KY , Staten LK , Gillespie C , Ranger-Moore J , Thomson CA , Giuliano AR , Ethnicity and nutrient intake among Arizona WISEWOMAN participants. J Womens Health (Larchmt) 2007;16(3):379–89. 10.1089/jwh.2006.M078 17439383

[5] Pérez-Escamilla R , Putnik P . The role of acculturation in nutrition, lifestyle, and incidence of type 2 diabetes among Latinos. J Nutr 2007;137(4):860–70. 17374645 10.1093/jn/137.4.860

[6] National Center for Health Statistics. About the National Health and Nutrition Examination Survey. http://www.cdc.gov/nchs/nhanes/about_nhanes.htm. Accessed May 2015.

[7] Guenther PM , Casavale KO , Reedy J , Kirkpatrick SI , Hiza HA , Kuczynski KJ , Update of the Healthy Eating Index: HEI-2010. J Acad Nutr Diet 2013;113(4):569–80. 23415502 10.1016/j.jand.2012.12.016 PMC3810369

[8] Kandula NR , Diez-Roux AV , Chan C , Association of acculturation levels and prevalence of diabetes in the Multiethnic Study of Atherosclerosis (MESA). Diabetes Care 2008;31(8):1621–8. 10.2337/dc07-2182 18458142 PMC2494621

[9] Thiele S , Mensink GB , Beitz R . Determinants of diet quality. Public Health Nutr 2004;7(1):29–37. 10.1079/PHN2003516 14972069

[10] Hiza HA , Casavale KO , Guenther PM , Davis CA . Diet quality of Americans differs by age, sex, race/ethnicity, income, and education level. J Acad Nutr Diet 2013;113(2):297–306. 10.1016/j.jand.2012.08.011 23168270

[11] Nicklas TA , Qu H , Hughes SO , He M , Wagner SE , Foushee HR , Self-perceived lactose intolerance results in lower intakes of calcium and dairy foods and is associated with hypertension and diabetes in adults. Am J Clin Nutr 2011;94(1):191–8. 10.3945/ajcn.110.009860 21525197

[12] Fulgoni VL 3d , Agarwal S , Spence L , Samuel P . Sodium intake in US ethnic subgroups and potential impact of a new sodium reduction technology: NHANES dietary modeling. Nutr J 2014;13(1):120. 10.1186/1475-2891-13-120 25522786 PMC4290401

